# Collaborative Development of a Simulation-augmented Health Education Program in Resource-challenged Regions

**DOI:** 10.7759/cureus.2850

**Published:** 2018-06-21

**Authors:** Tia S Renouf, Melanie Doyle, Megan Pollard, Tina Bankovic, Adam Dubrowski

**Affiliations:** 1 Emergency Medicine, Memorial University of Newfoundland/Tairawhiti Hospital, Gisborne, NZL; 2 Emergency Medicine, Memorial University of Newfoundland, Gisborne, NZL; 3 Emergency Medicine, Memorial University of Newfoundland, St. John's, CAN

**Keywords:** simulation, health professional education, hpe, global health

## Abstract

Simulation-based health professional education (HPE) is widely practiced in resource-rich regions, yet it is underutilized or ineffectively delivered in resource-poor ones, particularly when we fail to consider local contexts such as infrastructure, literacy, and culture when developing educational programs. Such an approach would be an example of failure of delivery, or the inability to bring services to people whose diseases have proven therapies. It is the biggest obstacle facing global health. This paper is a review of the literature and the authors’ experience in developing, delivering, and evaluating sustainable HPE programs in resource-poor regions, wherever in the world they may be.

## Introduction and background

Failure of delivery, or the inability to bring services to people whose diseases have proven therapies, is the biggest obstacle facing global health [1]. Although usually considered in clinical terms, failure of delivery also happens at systems levels [2] and with educational interventions. Even as increasing numbers of North American short-term medical missions aim to bring a much-needed medical care and health professional education (HPE) to resource-poor areas, they too often fail to deliver sustainable results, as they do not acknowledge local contexts and the systems that govern them. They may “dance to their own rhythms rather than to the tune of local priorities” [2].

It is appealing to combine humanitarianism with hands-on patient care, and opportunities are increasing for health professionals to embark on such missions [3]. However, the structure and organization of these missions may be inconsistent, [4] and their impact in resource-poor regions is controversial [5]. Visiting teams that fail to consider local contexts like infrastructure, literacy, and culture [6], strain local healthcare initiatives and may unintentionally undermine local healthcare providers [6]. Moreover, short-term missions are not commonly evaluated, have limited follow-up data, and may introduce practices that are unsustainable [4]. This may affect health performance systems, which in order to deliver, must be subject to holistic evaluation [7]. We need to understand how context features in this problem if we are to achieve the United Nations’ as yet unmet 2015 millennium health-related goals [8] and progress towards the 2030 Sustainable Development Goals. Health is integral to sustainable development; better health boosts educational and economic outcomes [9], and is linked with economic and social development [10]. It follows that health education plays a role.

Simulation is a globally applicable HPE intervention, used to teach a variety of medical skills and procedures. It is risk-free to patients, and as such is a moral health imperative [11]. If employed sustainably and at reasonable cost [12], it may help achieve the 2030 Sustainable Development Goals. While simulation-augmented HPE is effective in resource-rich areas [13], we limit its impact in resource-poor ones when we attempt to employ it with sophisticated, expensive, and difficult-to-maintain tools like computerized human mannequins. Early collaboration with local stakeholders—local experts—is essential in order to design applicable and sustainable education. Through its positive health impact, this collaboration may enable economic, educational, and professional growth in resource-poor areas. There are similar teaching challenges in our own (Canadian) rural and remote backyards where, for instance, local indigenous populations have poor health indices [14]. Perhaps these challenges will benefit from similar solutions.

This paper is a review of the literature and the authors’ experience in developing, delivering, and evaluating sustainable HPE programs in resource-poor regions. We present 12 key steps that may help achieve this goal.

## Review

First, know the local context. Brazilian philosopher and educator Paulo Freire said, “transformation is valid only if it is carried out with the people, not for them” [15]. Follow this advice and acknowledge that local literacy, infrastructure, and culture will significantly affect a program’s design and delivery. To ignore this advice risks failure of delivery, and invites both patient [16] and systemic [17] harm. Patients are harmed, for instance, when visiting medical teams dispense potentially toxic drugs to those who cannot read warning labels [18]. Local systems are harmed when well-meaning governments and NGOs donate unusable equipment to resource-poor regions.

The problem applies equally to educational programs. During a recent visit to Haiti, completing a simulation-based professional development exercise, we were shown an example of what could be called simulation-related systemic harm. While touring the hospital grounds, we learned that a shipping container full of unused equipment was generously donated by an NGO, meaning to form a modern emergency department, but was unusable because of electrical incompatibilities and the lack of available personnel with the necessary time or expertise to incorporate new system. Expensive high-fidelity simulators should not be sent to resource-poor regions without extensive local consultation. Thoroughly assess the equipment’s utility: Are the plugs and voltage compatible? How does the equipment react to environmental conditions? Can minor repairs be managed locally? Growing evidence suggests that many clinical tasks and skills are learned just as well from simple trainers as from complex human mannequins [19-20]. Under what circumstances, then, are donations of the latter a wise use of resources?

Second, conduct a needs assessment. Collaborate with local colleagues and carefully consider who the students are, what skills they need to learn, and what local teaching resources are available. Sophisticated simulation equipment is a highly valued investment that should come with a warning label: do not overestimate how well this tool applies to a resource-poor setting. Heed this warning and use a needs assessment tool to ask local educators what works for them [21]. Our Haitian colleagues, for instance, told us during our needs assessment, that they had already built their own task trainers and did not need sophisticated simulation equipment, nor could they support it. They showed us we had the wrong approach, and compelled us to tailor our initial program goals from: 1) enable local educators to develop their own simulation resources and 2) learn about underlying educational principles, to: 1) enable local educators to use human models to contextualize their simulators, and 2) develop a cascading train-the-trainer approach so local educators can both learn and teach others. Working with local professionals to conduct a needs assessment prevented us from delivering the wrong curriculum.

We used two simple needs assessment tools to develop our collaborative program: SWOT (Strengths, Weaknesses, Opportunities, and Threats) [22] and CIPP (Context, Input, Process, and Product) [23]. We used SWOT to specify our program’s objectives, and to identify internal and external factors that would either help us achieve them, or stand in our way. Next, we used CIPP to systematically develop the program while we gathered broad information about its development, improvement, management, and operation. Similar tools can be used to develop program content that both delivers expected results and flags unexpected ones. Enable your hosts to design educational programs that address their region’s own most pressing health and education priorities.

Finding a local champion is our third step. The educational program is more likely to succeed if visitors collaborate with a local project champion who leads it from start to finish [24]. He or she may be a respected leader or educator, passionate about improving local health outcomes, who can drive innovative organizational change [24]. The project champion may be well placed to help avoid failure of delivery if they grew up in a resource-poor region and witnessed or experienced it. He or she will use local knowledge to work around obstacles that a visiting educator may not even see.

A culturally relevant reward may be an effective incentive for your project champion when, as is often the case, no salary is available. For example, one of the authors identified potential titles for two project champions working at Addis Ababa’s Black Lion Hospital Simulation Centre in Ethiopia. These unpaid educators’ leadership and commitment were remunerated with the positions of Director and Patron; both individuals became instrumental in rapidly expanding the hospital’s simulation program.

Fourth, align educational goals with your partners’, and understand that the program belongs to them. Clearly establish each collaborator’s contribution to the program, and iteratively review, reinforce, and modify the plan when necessary. Careful examination and reflection by all parties is essential, both before the project starts and as it unfolds. Be clear that everyone understands the program’s objectives, and agrees that local ownership is the goal.

Strengthen this approach with a memorandum of understanding, or an agreement among collaborating parties. Although the document may hold little or no legal value, it states common goals and charts a course for achieving them. Understand that novel ideas must eventually become organized [25], and that the local approach is valuable; it is likely to be sustainable if it has evolved in response to its context.

Fifth, garner support from all angles: administrative, collaborative, and financial. Administrative leaders can lend support if they want you to invest in training local health professionals [26]. Two such individuals supported us in Haiti. One (the Chief of Staff) indicated his interest in collaborating on the day we first met. He formed a highly motivated steering committee, whose members informed the second administrative leader (the CFO) that simulated training environments are cheaper for teaching than over-burdened public hospitals [27]. This motivated the CFO to seek funds for a dedicated teaching space. Success was infectious; we returned the following year with a Haitian-specific simulation curriculum, and with the help of local colleagues, delivered it to a room full of supremely engaged learners and faculty. The CFO periodically interrupted his busy day to check in and support our participants.

Top-down initiatives also need bottom-up collaborations to provide soil for young roots [28]. A top-down oriented medical team, for example, must collaborate and integrate with local health providers and systems. The same is true for educational programs. Failure to integrate can result in failure of delivery [4]. Include the skeptics! Those who disagree with the project’s intent can help identify its flaws. Be observant; ask yourself who stands to gain or lose from the initiative.

Seek financial support for the program. Recently, public and private sectors have collaborated to achieve a common objective: benefit that does not compromise public interest [29]. Thus, show measurable outcomes to potential funders, explain and quantify how well-designed, targeted health education expenditure is an investment, and demonstrate how health education programs facilitate national development objectives [30].

Financial support signals that the project is highly regarded. For example, a peer-reviewed grant presents the project to a broad academic community. Funding also establishes accountability, sets timelines, and supports travel, enabling participants from resource-rich and -poor regions to experience one another’s contexts. It also supports salaries and essential equipment. Based on our collective experience, even projects with small awards are more successful than those with no funding at all.

Sixth, realize that a fast pace may slow you down in resource-poor regions. Culturally diverse team members may speak different languages. They may also have different perceptions of the local hierarchy, and choose to navigate it in unfamiliar ways. Although visitors may be comfortable questioning leaders, doing so is considered disrespectful, for instance, in some Asian cultures [31]. Approach these nuances carefully with the project champion; balance them with the team’s need for candid communication [32]. Also, listen to colleagues, learn from their diversity, and be attuned to any dissonance emerging from different cultural points of view.

While in Haiti, for example, we delivered a simulation workshop with the local educators. While we delivered our lectures, we were unprepared for our audiences’ constant influx and egress through a squeaky swinging door and for their continuous cell phone use. Our participants clearly had other work to do, now. This behaviour may have slowed us down, but it sped them up.

Seventh, use materials local to where you are teaching. For example, we taught chest tube placement in a remote part of our own Canadian backyard. We built our task trainer with hard foam from a construction site and pork ribs from a local grocer. We mounted it on a banker’s box and draped it all in surgical cloth. The local educators taught the procedure with their own thoracentesis kits. Further afield in Ethiopia, participants in one study used shoelaces to practice their surgical throws [33]. Shoelaces are ubiquitous and do not dry out and break over time like commercial sutures. Local markets overflow with this kind of raw material. Mine through them and find it.

Local markets are also a vibrant living display of the social determinants of health. Send local and visiting learners there together, and see if they bring back a better mutual cultural understanding along with their raw material. Use simulators to teach locally relevant practices, and remember that inappropriate teaching tools are harmful when used in conditions of privation. They divert busy educators from delivering more sustainable education.

Eighth, use local knowledge. Knowledge Translation (KT) is a scholarly approach that can translate the program’s findings into practical local knowledge that feeds back into and improves the program [2]. Translate your knowledge with diverse local organizations like hospitals, universities, and patient groups. Further, circulate it between resource-rich and -poor regions. Our Haitian colleagues did this when visiting several Canadian academic simulation centres. They experienced our educational practices, took them home, and modified them, with plans to translate their new Haiti-specific knowledge for their own policy makers, health professionals, and patients. Earlier we described how our Haitian and Ethiopian partners improved their programs by making task trainers solely with locally available materials. These kinds of modifications, used alongside candid program assessment, will help to integrate the syllabus locally.

Local knowledge is the program’s bedrock; publish and disseminate it. Co-authorships will help illuminate your partners’ scholarly work, as will publishing in the local language. Some international academies feature local work and raise its visibility by linking up with smaller journals [16]. Visibility is helpful, but resource-poor regions also need universal access to educational tools. For example, the Canadian Tri-Council now mandates that its grant recipients make their work broadly accessible through open-access publications [34]. With similar intent, we recently published a collaborative scholarly work featuring Haitian-made simulation trainers. We chose an open-access journal in order to make it accessible for health professionals in low resource contexts [35].

Ninth, train more trainers. Simulation-based teaching takes time and effort [36]; this is burdensome for the few trained educators who work in resource-poor regions [37]. Help to lighten this load by collaboratively Training-the-Trainer (TTT). Earlier we described how Ethiopian researchers used shoelace sutures to teach simple surgeries; that study came about because of a shortage of both teachers and health workers [38]. The study’s goal was to cascade surgical training to even more trainers, thereby reducing the demand for faculty, while simultaneously filling a clinical gap. Pairing senior learners with novices promotes active learning and retention for both [39], and also enhances collaboration, confidence, and professionalism [40]. Junior learners are satisfied with the simulation debriefs that senior learners provide [41]. In another Ethiopian initiative, Canadian and British educators teamed up with local health providers to study that country’s high maternal and infant mortality rates. Together they designed the Ethiopian Training of Trainers (TOT) program that over several years, trained sequential cohorts of trainers to practice and teach surgery and obstetrics and gynecology, along with more general clinical skills. The program cascaded out rurally from its urban origin [38].

Tenth, anticipate consequences. Projects are unlikely to evolve predictably. Be attuned to this and remain flexible [42]. Identify avenues within the partnership from which participants may gain power and privilege, and anticipate the social and cultural damage that may ensue. Some requests may seem puzzling, such as the provision of on-site housing for local educators. While this may seem to lie outside the project’s mandate [17], local educators might have domestic obligations and lack reliable transportation. The project champion will understand these kinds of dynamics, and may also know ways to reignite waning interest. For instance, local leaders can encourage their junior staff by giving them titles and responsibilities, and together you can consider adjunct academic appointments at your respective universities [26]. Develop and nurture close, trusting relationships among partners in order to prevent attrition; losing interest in an abstract program is harder if it means letting down a respected colleague.

The eleventh step is to fade away. Aggressively and persistently plan for independence. Enable local program control once the learning environment is strong enough to support the new knowledge and skills. Although visits between regions are desirable, avoid contributing to the "brain drain" that may come with internationally recognized professional credentials. Teach locally relevant skill sets; unused ones are likely to wither. Ideally, the collaborations built among administrative leaders, the local champion, and both teams, working from the aforementioned solid structural foundations, will leave a sustainable and well-integrated project. Fading away does not mean disappearing altogether; withdrawal plans should include long-distance support and periodic reassessments [43].

Finally, evaluate the program. William Boyd (1990) wrote: “The last thing we learn about ourselves is our effect” [44]. So it is with our educational programs. Only holistic evaluation will illuminate the program’s strengths and weaknesses; only iterative reconstruction will prevent failure of delivery. Complex human interventions do not operate in a vacuum; they nest instead alongside concepts of equity and justice, surrounded by complex notions of culture and social determinants of health [45]. These will all factor into the program evaluation, an uncommon practice whose value some organizations are fortunately now be­ginning to recognize [45-46].

Evaluation models have evolved over time; they once simply judged whether a program fulfilled its designer’s objectives. Roadmaps now take a more nuanced look at how a syllabus operates within its unique environment [42]. They aim to discover how and why a program works, and to reveal its unpredicted outcomes. Collaborate with local educators and feed both predicted and unpredicted outcomes back into what you have created together. The Ethiopian Patron position we described earlier came to be when a program evaluation revealed that hierarchical inertia had stalled forward progress. Such feedback will help iron out wrinkles and improve program sustainability.

## Conclusions

Health inequities and limited access to health education exist in resource-poor areas. Simulation-augmented HPE is a moral health and educational imperative, one that can help us achieve the 2015 Millennium Goals and the 2030 Sustainable Development Goals if we collaboratively spread it to the resource-poor regions both in developing countries and our own backyards. We describe a way to develop and deliver a sustainable simulation program to resource-challenged regions, based on both a review of the literature and the authors’ experience.
